# Derecruitment Test and Surfactant Therapy in Patients with Acute Lung Injury

**DOI:** 10.1155/2012/428798

**Published:** 2012-08-15

**Authors:** Alexey A. Smetkin, Vsevolod V. Kuzkov, Konstantin M. Gaidukov, Lars J. Bjertnaes, Mikhail Y. Kirov

**Affiliations:** ^1^Department of Anesthesiology and Intensive Care Medicine, Northern State Medical University, Troitsky Avenue 51, Arkhangelsk 163000, Russia; ^2^Department of Anesthesiology and Intensive Care Medicine, City Hospital No. 1 of Arkhangelsk, Suvorov Street 1, Arkhangelsk 163001, Russia; ^3^Department of Clinical Medicine (Anesthesiology), Faculty of Medicine, University of Troms, MH-Breivika, 9038 Troms, Norway

## Abstract

*Introduction*. A recruitment maneuver (RM) may improve gas exchange in acute lung injury (ALI). The aim of our study was to assess the predictive value of a derecruitment test in relation to RM and to evaluate the efficacy of RM combined with surfactant instillation in patients with ALI. *Materials and Methods*. Thirteen adult mechanically ventilated patients with ALI were enrolled into a prospective pilot study. The patients received protective ventilation and underwent RM followed by a derecruitment test. After a repeat RM, bovine surfactant (*surfactant group*, *n* = 6) or vehicle only (*conventional therapy group*, *n* = 7) was instilled endobronchially. We registered respiratory and hemodynamic parameters, including extravascular lung water index (EVLWI). *Results*. The derecruitment test decreased the oxygenation in 62% of the patients. We found no significant correlation between the responses to the RM and to the derecruitment tests. The baseline EVLWI correlated with changes in SpO_2_ following the derecruitment test. The surfactant did not affect gas exchange and lung mechanics but increased EVLWI at 24 and 32 hrs. *Conclusions*. Our study demonstrated no predictive value of the derecruitment test regarding the effects of RM. Surfactant instillation was not superior to conventional therapy and might even promote pulmonary edema in ALI.

## 1. Introduction

Acute lung injury (ALI) is associated with significant morbidity and mortality in critically ill patients [1–3]. Several mechanisms are involved in the development of ALI. The enhanced pulmonary capillary leakage causing pulmonary edema is one of the key factors. Another important mechanism is the formation of atelectases secondary to depletion of surfactant and accumulation of lung tissue fluid [4–6]. The latter mechanisms in combination with derangement of hypoxic pulmonary vasoconstriction may cause increased venous admixture and progressive deterioration of oxygenation [4, 7, 8].

The recruitment maneuver (RM) is a widely used technique aiming to reopen atelectatic lung areas in patients with ALI. Transient increase in the airway pressure up to 40–60 cm H_2_O for 40–60 sec reexpands the deaerated lung tissue areas and improves oxygenation [9–11]. However, the influence of RM on the outcome is controversial [12, 13]. Moreover, RM has a number of adverse effects; the most significant of those are barotrauma and cardiovascular collapse [14–17]. The risks of RM are justified predominantly in potential responders, necessitating a search for tests that can predict the response to the maneuver.

The airway suctioning procedures require deliberate disconnection of airway tubes thereby reducing PEEP to 0 cm H_2_O. This may lead to alveolar derecruitment that produces effects opposite to those of the RM [18]. The most prominent of these effects are reduction of lung compliance and significant decrease in arterial oxygenation. We hypothesized that changes in oxygenation and lung mechanics at the time of derecruitment may be dependent on the antiatelectatic potential related to the response to RM, and thus, prevent its use in potential nonresponders.

Depletion of surfactant is an important factor predisposing to formation of atelectases and alveolar consolidation in patients with ALI. In patients with surfactant deficiency and alveolar instability, RM may be followed by hypoxemia and rapid reconsolidation of lung tissue [19]. Taking this theoretical background into account, the combination of RM and surfactant therapy could be of potential benefit in patients with ALI.

Therefore our study had two goals: (1) to explore whether the efficacy of RM might be predicted on the basis of changes in oxygenation and lung mechanics provoked by the derecruitment maneuver, and (2) to assess the effects of RM combined with endobronchial instillation of surfactant in patients with ALI.

## 2. Materials and Methods

The study was conducted in compliance with the Helsinki Declaration. The study protocol and the informed consent form were approved by the Ethics Committee of Northern State Medical University, Arkhangelsk, Russian Federation. Written informed consent was obtained from every patient or next of kin.

Thirteen adult patients with ALI requiring mechanical ventilation (MV) were enrolled into the pilot study. All the patients met the ALI criteria of the American-European Consensus Conference [20]. Exclusion criteria were anticipated duration of MV of less than 24 hours, duration of ALI more than 24 hours before the start of study, and inability to perform alveolar recruitment maneuver due to comorbidities. The severity of illness at the entry of study was estimated using SAPS II score. The severity of organ dysfunction and lung injury were assessed at the start of study and at 24 and 48 hrs employing the SOFA score and the lung injury score (LIS), respectively. Patients were sedated with fentanyl (1 mcg/kg/hr) and midazolam (0.05 mg/kg/hr) and ventilated using pressure-controlled ventilation (Avea, VIASYS Heathcare, USA) with the following baseline ventilator settings: tidal volume 7 mL/kg of predicted body weight, FiO_2_ 0.5, and PEEP 4 cm H_2_O. The respiratory rate was adjusted to maintain PaCO_2_ of 35–45 mm Hg. If these settings did not result in a SaO_2_ ≥ 92%, FiO_2_ was increased by steps of 0.1 every two minutes up to 0.8.

We recorded parameters of mechanical ventilation including tidal volume (*V*_*T*_), inspiratory oxygen fraction (FiO_2_), peak airway pressure (*P*_peak_), mean airway pressure (*P*_mean_), positive end-expiratory pressure (PEEP), and respiratory compliance (*C*). In parallel, we analyzed arterial blood gases including pH, PaCO_2_, PaO_2_, SaO_2_, base excess (BE), and lactate concentration. The end-tidal CO_2_ (EtCO_2_) was registered using Capnostream TM monitor (Oridion, Israel).

All the patients were cannulated with a 5 F femoral artery catheter (Pulsiocath PV2015L20, Pulsion) and an 8.5 F jugular central venous catheter (triple-lumen 20 cm catheter). The hemodynamic monitoring was performed using the single transpulmonary thermodilution technique with PiCCO_2_ monitor (Pulsion Medical Systems, Germany). The thermodilution measurements were performed in triplicate with injections of ice-cold (<8°C) 5% dextrose solution via a central venous catheter. The registered hemodynamic parameters included central venous pressure (CVP), cardiac index (CI), mean arterial pressure (MAP), stroke volume index (SVI), systemic vascular resistance index (SVRI), heart rate (HR), global ejection fraction (GEF), left ventricle contractility index (dPmx), global end-diastolic volume index (GEDVI), and extravascular lung water index (EVLWI) adjusted to predicted body weight (PBW), which was calculated as follows: PBW (kg) = 50 + 2.3 (height (cm)/2.54–60) for male, and PBW (kg) = 45 + 2.3 (height (cm)/2.54–60) for female.

### 2.1. Recruitment Maneuver

After baseline measurements and muscular blockade with pipecuronium (0.06 mg/kg), RM was performed by applying a continuous positive airway pressure of 40 cm H_2_O for a period of 40 seconds [9, 21]. The recruitment maneuver was discontinued in case of hypotension (MAP < 50 mm Hg, or a decrease in MAP by more than by 30 mm Hg from the initial value), or hypoxemia (SpO_2_ < 85% or a decrease by more than 10%). Then, pressure-controlled ventilation was resumed with the same settings as before RM. The level of PEEP was set at 2 cm H_2_O above the lower inflection point (LIP) of the pressure-volume (*P*-*V*) curve determined by an inflection point maneuver of the ventilator, but not less than 4 cm H_2_O.

The arterial blood gases, SpO_2_, *V*_*T*_, and EtCO_2_ were registered at 5 min after the RM. The efficacy of the RM was assessed by detecting the changes in SpO_2_ and *V*_*T*_. Patients were defined as responders if the absolute SpO_2_ value increased by at least 2% or *V*_*T*_ rose by at least 10% [10]. The stability of the RM was assessed by registering the changes in SpO_2_, *V*_*T*_, and EtCO_2_ at 5 min intervals during the subsequent 30 min period. The RM was considered as stable if the absolute value of SpO_2_ decreased by ≤2% or *V*_*T*_ decreased by ≤5%.

### 2.2. Derecruitment Test

After the assessment of RM stability, the derecruitment test was performed. Positive end-expiratory pressure was set at 0 cm H_2_O for a period of 15 minutes. Other parameters of mechanical ventilation were unchanged. The changes in SpO_2_, *V*_*T*_, and EtCO_2_ were registered every five minutes. At the end of the derecruitment test, we analyzed the arterial blood gases. The derecruitment test was interrupted in case of severe hypoxia (SpO_2_ < 85%). The test was defined as positive if it resulted in a decrease in SpO_2_ by at least 2% and in *V*_*T*_ by at least 10%, respectively.

### 2.3. Surfactant Therapy

After the derecruitment test, PEEP was adjusted to the previous value. The patients were randomized, by means of the sealed envelope method, to a surfactant therapy group (ST group, *n* = 6), and a conventional therapy group (CT group, *n* = 7). Physicians and research staff were not blinded to the study groups. The ST group received the surfactant emulsion (Surfactant-BL, Biosurf, Russia) prepared *ex tempore* and administered into the segmental bronchi in a total dose of 6.0 mg/kg of PBW (0.4 mL/kg of PBW) by means of fiberoptic bronchoscopy (FOB) according to the recommendations of the manufacturer. The CT group received an equivalent volume of 0.9% NaCl endobronchially. After FOB and instillation of surfactant emulsion or 0.9% NaCl, the RM was repeated followed by adjustment of PEEP. The FOB with instillation of the study medicine was repeated at 18 hrs and 32 hrs.

Arterial blood gases and parameters of hemodynamics and mechanical ventilation were registered at 1, 2, 4, 8, 16, 24, 32, and 48 hrs after the initial instillation of surfactant emulsion or 0.9% NaCl.

### 2.4. Statistical Analysis

For data collection and analysis we used SPSS software (version 18.0; SPSS Inc, Chicago, IL). The data distribution was assessed with Shapiro-Wilk test. Quantitative data were presented as mean ± standard deviation or median (25th–75th percentile) depending on the data distribution. The discrete data were expressed as absolute values or percentages. In case of normal distribution, we used Student's *t*-test for comparisons between groups. Nonparametrically distributed data were assessed by the Mann-Whitney *U*-test. The correlation analysis was performed using Pearson's or Spearman's tests for parametrically and nonparametrically distributed data, respectively. The discrete data were evaluated using chi-square test. For all tests, a *P* value < 0.05 was considered as significant.

## 3. Results

The individual demographic and clinical characteristics of the patients are presented in Table 1. At the study entry, patients had a mean SAPS II score of 40 ± 13 points and a mean SOFA score of 8.7 ± 3.0 points. During the study, the severity of organ dysfunction decreased slightly to SOFA score of 7.4 ± 3.3 at 48 hrs. Initially, the patients had severe lung injury accompanied by a LIS of 2.5 ± 0.7 points. During the study, LIS did not change significantly. The severity of organ dysfunction and lung injury did not differ between the study groups.

In response to the RM, the changes in SpO_2_ correlated with the changes in PaO_2_ (*r* = 0.79, *P* < 0.01). As evaluated by the changes in SpO_2_, the RM was successful in 62% of the patients. In parallel, the RM increased the tidal volume significantly (>10%) in 31% of the patients.

The assessment of the stability of the RM revealed a significant decrease in SpO_2_ among 50% of the responders and a decline in *V*_*T*_ in 70% of the responders to RM.

The derecruitment test resulted in a decrease in SpO_2_ in 62% of the patients and a reduction of *V*_*T*_ in 54% of the patients. During the derecruitment test, SpO_2_ decreased in 71% of the responders and 50% of the nonresponders. Most of the patients presented with SpO_2_ ≥ 90%. In three patients, the derecruitment test was interrupted within 5 min due to a rapidly developing hypoxemia. Following derecruitment, a reduction of *V*_*T*_ was revealed in 100% of the responders to RM and 38% of the nonresponders. We found no correlations between the changes in SpO_2_ and *V*_*T*_ in response to the RM or to the derecruitment test. The changes in PaO_2_ after RM correlated inversely with changes in *V*_*T*_ during the derecruitment test (*r* = −0.72, *P* < 0.05).

We found no significant differences regarding the effects of RM and the derecruitment test in patients with direct and nondirect ALI.

The baseline EVLWI correlated with changes in SpO_2_ during the derecruitment test (*r* = 0.7, *P* < 0.05), but did not correlate with changes in *V*_*T*_.

The changes in volumetric parameters, blood gases and lung mechanics are presented in Table 2. After performing the tests and at 16 hrs, PaCO_2_ was significantly higher in the ST group. The surfactant therapy did neither affect PaO_2_/FiO_2_, PaCO_2_, and EtCO_2_, nor minute volume of ventilation, respiratory compliance, selected PEEP values, or FiO_2_. However, the ST group demonstrated a significant increase in EVLWI at 24 and 32 hrs.

## 4. Discussion

Our study demonstrated no predictive value of the derecruitment test regarding its possibility to uncover effects of RM in patients with ALI. This necessitates a search for alternative predictors of the response to alveolar recruitment. Moreover, the endobronchial instillation of surfactant was not superior to conventional therapy in patients with ALI, and our study revealed that it might even worsen the development of pulmonary edema.

### 4.1. Recruitment and Derecruitment

The changes in SpO_2_ we noticed during RM correlated with the changes in PaO_2_. This allowed us to assess oxygenation continuously by means of SpO_2_, which is more readily available at the bedside, as compared to frequent blood gases analyses. Identifying responders to RM, we used the cut-off value of SpO_2_ ≥ 2%, which is the average increase in SpO_2_, as demonstrated by *The ARDS Clinical Trials Network* [10]. In contrast to SpO_2_, *V*_*T*_ increased only in one-third of the patients. Moreover, the recruitment effects were unstable in the majority of patients. These findings may be explained by a prevalence of direct lung injury due to pneumonia (77% of the studied patients) that demonstrate less effective recruitment maneuver and a predisposition to formation of atelectases [21–23].

The high rate of desaturation and pulmonary recollapse following the derecruitment test reflect changes that can be observed in response to airway disconnection. The lack of a predictive value of the supposed derecruitment test might be explained by the different pattern of changes in ventilation-perfusion interaction during alveolar recruitment and derecruitment [24, 25]. However, the inverse correlation between the changes in PaO_2_ after the RM and the changes in *V*_*T*_ during the derecruitment test demonstrates that patients with lack of improvement of oxygenation during the RM are more predisposed to recollapse of alveoli during the derecruitment. It might be explained by a predominance of the mechanisms of lung consolidation rather than by pulmonary edema in this group of patients. This speculation corresponds to the absence of correlation between changes in *V*_*T*_ during desaturation and the baseline value of EVLWI. In contrast, the correlation between the baseline EVLWI and the severity of desaturation during the derecruitment test demonstrates a predisposition of patients with lung edema to more severe hypoxemia following derecruitment. These results confirm the potential risk of airway disconnection and should be considered during tracheal suctioning of patients with ALI.

### 4.2. Surfactant Therapy

Our study demonstrated a lack of effect of surfactant therapy in combination with RM in patients with ALI. As evidenced by a recent metaanalysis, these findings are consistent with the results of most of the previous studies in this field [26]. An explanation of the negative result could be that administration of surfactant took place in patients in whom pulmonary edema already had developed, as confirmed by increased EVLWI. We cannot exclude the possibility that alveolar fluid might have inactivated both endogenously produced and exogenously administered surfactant. On the other hand, it could be potentially harmful to restore an assumed lack of surfactant without preassessment of the actual deficit. Therefore, further investigations are required with special focus on the efficacy of surfactant replacement therapy in patients with confirmed surfactant insufficiency.

Although the surfactant treatment used in this study did not influence lung mechanics or alveolar gas exchange, it unexpectedly resulted in enhancement of lung edema. In a recent study by Lu et al. using computed tomography, it has been shown that instillation of exogenous surfactant in patients with ALI/acute respiratory distress syndrome (ARDS) caused substantial expansion of nonaerated lung areas [27]. Several mechanisms might be involved in the progression of lung edema. One of the possible mechanisms is the retention of lung water by hydrophilic components of the surfactant proteins. Another mechanism could be an inflammatory reaction, which is evoked by an interaction between the exogenously administered surfactant and the active endogenous surfactant resulting in capillary leakage and increase in lung edema [28]. Last but not least, improvement of lung tissue aeration and attenuation of pulmonary hypoxic vasoconstriction may extend the contact area between the thermal indicator and the pulmonary vascular bed leading to increase in the measured EVLWI value [29].

Our study has several limitations including a small sample size, heterogeneous patient characteristics, a relatively high prevalence of patients with direct lung injury, and nonblinded treatment with bovine surfactant. The power analysis performed before our study and based on our hypothesis, that surfactant could lead to a 30% increase in PaO_2_/FiO_2_ with no changes in PaO_2_/FiO_2_ in the CT group, revealed that assuming a two-sided *P* value of 0.05 and 80% power, a sample size of 18 patients in each group is required. However, after analysis of our pilot results, we stopped the study prematurely because we found no beneficial effects of the surfactant therapy. Despite we displayed a significant correlation between the changes in SpO_2_ and the changes in PaO_2_, the use of changes in SpO_2_ instead of PaO_2_/FiO_2_ for assessment of the efficacy of RM may also be a limitation of our study. Thus, the results of this study as well as the use of derecruitment test and the surfactant therapy in ARDS require further investigation in larger clinical trials.

## 5. Conclusions

In ALI, the derecruitment test appears to have no predictive value in terms of assessing a potential effect of the alveolar recruitment maneuver. Surfactant therapy combined with RM does not seem to provide any further benefit in comparison with RM and conventional therapy and may even promote lung edema in patients with ALI or ARDS.

## Figures and Tables

**Table 1:** Demographic and clinical characteristics of patients with acute lung injury.

Patient	Group	Diagnosis	Age, years	Gender	Height, cm	Weight, kg	SAPS II, points	SOFA, points	LIS, points	PaO_2_/FiO_2_, mm Hg	PaCO_2_, mm Hg
1	ST	Pancreatitis	56	m	170	75	30	5	1.70	268	48.9
2	ST	Pneumonia	68	m	171	77	48	9	1.50	185	43.4
3	ST	Pneumonia	57	m	175	80	46	8	2.25	155	50.5
4	CT	Pneumonia	66	m	162	60	44	9	1.75	200	32.9
5	CT	Pneumonia	33	m	180	70	37	12	2.75	73	44.4
6	CT	Peritonitis	78	f	162	85	66	9	1.75	240	41.7
7	ST	Pneumonia	25	m	183	70	25	6	3.67	121	48.6
8	CT	Pneumonia	57	m	175	75	46	11	2.00	125	49.9
9	CT	Pneumonia	31	f	170	86	22	4	3.25	73	45.4
10	ST	Fat embolism	27	m	178	75	28	5	2.75	189	41.7
11	ST	Pneumonia	52	m	176	90	31	10	3.25	45	55.7
12	CT	Pneumonia	51	m	175	78	45	14	2.50	71	57.0
13	CT	Pneumonia	72	m	175	120	54	11	3.00	83	42.7

Data are presented as absolute values.

ST: surfactant therapy; CT: conventional therapy; LIS: lung injury score; m: male; f: female.

**Table 2:** Changes in hemodynamics, arterial blood gases, and lung mechanics in patients with acute lung injury.

Parameter	Group	After the tests	After the FOB	1 hr	2 hrs	4 hrs	8 hrs	16 hrs	24 hrs	32 hrs	48 hrs
CI, L/min/m^2^	ST	5.03 ± 1.04	4.77 ± 1.16	5.05 ± 1.10	4.79 ± 1.20	4.98 ± 1.15	4.99 ± 1.35	4.89 ± 1.16	4.62 ± 1.03	4.76 ± 0.93	4.35 ± 0.74
	CT	3.85 ± 1.84	3.90 ± 1.86	3.66 ± 2.09	3.80 ± 1.88	4.20 ± 2.37	3.96 ± 1.82	3.63 ± 1.81	2.75 ± 0.83	3.23 ± 1.10	3.42 ± 1.23
GEDVI, mL/m^2^	ST	863 ± 180	690 ± 88	743 ± 206	799 ± 243	797 ± 218	787 ± 142	752 ± 173	743 ± 207	800 ± 275	714 ± 89
	CT	694 ± 131	675 ± 149	714 ± 172	755 ± 156	749 ± 145	727 ± 111	676 ± 140	694 ± 84	660 ± 108	710 ± 97
EVLWI, mL/kg PBW	ST	19 ± 6	23 ± 5	17 ± 6	18 ± 8	17 ± 5	18 ± 4	19 ± 4	18 ± 5^a^	18 ± 3^a^	18 ± 5
	CT	14 ± 4	15 ± 5	14 ± 6	16 ± 7	14 ± 6	13 ± 6	15 ± 8	11 ± 2	10 ± 3	11 ± 5
PaO_2_/FiO_2_, mm Hg	ST	157 ± 62	142 ± 41	131 ± 49	159 ± 58	168 ± 71	147 ± 45	162 ± 126	146 ± 32	219 ± 98	189 ± 41
	CT	119 ± 40	154 ± 79	139 ± 50	142 ± 46	162 ± 76	168 ± 75	188 ± 89	196 ± 99	205 ± 84	195 ± 107
PaCO_2_, mm Hg	ST	56 ± 9^a^	58 ± 9	53 ± 8	51 ± 7	49 ± 11	50 ± 11	54 ± 8^a^	50 ± 15	44 ± 11	47 ± 17
	CT	45 ± 6	44 ± 11	42 ± 10	44 ± 11	41 ± 11	41 ± 11	40 ± 6	40 ± 3	41 ± 3	43 ± 4
EtCO_2_, mm Hg	ST	44 ± 7	45 ± 7	43 ± 7	42 ± 6	40 ± 5	40 ± 6	39 ± 5	40 ± 11	38 ± 9	41 ± 12
	CT	33 ± 10	37 ± 11	34 ± 11	33 ± 9	32 ± 8	34 ± 8	33 ± 7	31 ± 6	33 ± 6	37 ± 6
FiO_2_	ST	0.58 ± 0.20	0.63 ± 0.25	0.70 ± 0.22	0.63 ± 0.15	0.62 ± 0.13	0.58 ± 0.13	0.64 ± 0.16	0.59 ± 0.08	0.56 ± 0.08	0.50 ± 0.06
	CT	0.67 ± 0.14	0.70 ± 0.18	0.63 ± 0.11	0.63 ± 0.11	0.62 ± 0.11	0.59 ± 0.11	0.58 ± 0.10	0.54 ± 0.05	0.56 ± 0.08	0.55 ± 0.04
PEEP, cm H_2_O	ST	9 ± 6	11 ± 6	12 ± 7	10 ± 6	10 ± 6	10 ± 7	10 ± 5	11 ± 6	11 ± 6	9 ± 4
	CT	9 ± 4	8 ± 4	9 ± 5	10 ± 5	10 ± 5	9 ± 4	9 ± 4	9 ± 3	9 ± 4	9 ± 4
Compliance, mL/cm H_2_O	ST	33 ± 6	33 ± 6	34 ± 5	36 ± 8	37 ± 8	34 ± 8	34 ± 2	31 ± 3	35 ± 3	38 ± 7
	CT	28 ± 7	32 ± 8	31 ± 6	28 ± 5	28 ± 5	30 ± 7	31 ± 9	30 ± 7	32 ± 9	30 ± 7

Data are presented as mean ± standard deviation. ^a^intergroup difference (*P* < 0.05).

After the tests: after both recruitment maneuver and de-recruitment test; ST: surfactant therapy; CT: conventional therapy; FOB: fiberoptic bronchoscopy; CI: cardiac index; GEDVI: global end-diastolic volume index; EVLWI: extravascular lung water index; PBW: predicted body weight; PEEP: positive end-expiratory pressure.
